# Early Outcome of Simultaneous Bilateral Total Knee Arthroplasty Through the Oxford Knee Score in a Developing Country: A Prospective Cohort

**DOI:** 10.7759/cureus.65563

**Published:** 2024-07-28

**Authors:** Muhammad Ahsan Sulaiman, Muhammad Ahmed Ghazni, Muhammad Omer Farooq, Muhammad Arbaz Arshad Khan, S.M. Nabeel Noor, Pervaiz Hashmi

**Affiliations:** 1 Orthopedics, Aga Khan University Hospital, Karachi, PAK

**Keywords:** mean improvement in oxford knee score, knee replacement, knee osteoarthritis/ koa, oxford knee score, simultaneous bilateral knee arthroplasty

## Abstract

Introduction

The most common degenerative joint disease in adults is osteoarthritis. The gold standard treatment option for this aging society with greater functional demands is total knee arthroplasty. The Oxford Knee Score (OKS) assesses factors such as stiffness, pain, function, satisfaction, and quality of life, allowing you to quantify treatment success after surgery. According to published research, there isn't a perfect postoperative timepoint to use the OKS to obtain TKA outcomes. Therefore, at the time of evaluation, the OKS should reflect the best possible outcome for the patient group. This study was conducted to see the OKS in patients who underwent simultaneous bilateral knee replacement at six weeks and six months postoperatively and to see if there was a clinically significant difference in the mean OKS.

Methods

This prospective cross-sectional study was conducted at the Section of Orthopedic Surgery at Aga Khan University Hospital, a tertiary care center in Karachi, Pakistan. Patients who underwent simultaneous bilateral total knee replacement from October 2023 till December 2023 were included; exclusion criteria included patients who had a recent knee infection and extensor mechanism disruption. OKS was calculated at six weeks and six months postoperatively.

Results

The total number of patients included in the study was 49 with a mean age of 61.9 +/- 6.1. There were 42 (85.7%) females and 7 (14.3%) males. The mean BMI of our patients was 33.3 +/- 3.8. The radiographic Kellgren Lawrence Grading (KLG) was used and 38 (77.6%) patients were placed in Grade IV KLG, and 11 (22.4%) were placed in Grade III KLG. The mean OKS preoperatively was 12.6 +/- 3.5. At six weeks, the OKS showed improvement, with the mean being 20.6 +/- 3.0. At six months postoperatively, there was a significant improvement in the OKS, with the mean now being 42.7 +/- 2.4. At six weeks post-surgery, the mean improvement in the OKS score was 7.9 +/- 2.71, whereas at six months post-surgery, the mean improvement in the OKS score was 30.1 +/- 3.6. This difference was significant (*p*-value=0.03).

Conclusion

Our study showed a clinically significant difference between the mean OKS at the six-week and six-month timeline, with a significant increase in the mean improvement OKS score at six months. OKS should be utilized six months postoperatively to assess the outcome of simultaneous bilateral knee arthroplasty patients.

## Introduction

The most common degenerative joint disease in adults is osteoarthritis (OA), affecting over 25% of the global adult population [1]. The 2013 Global Burden of Disease Study revealed that Pakistan, a developing country, has a prevalence of OA of 26.67 per 1,000 people [2]. This disabling condition, which is linked to age, obesity, genetic predisposition, and injury/inflammation, is expected to be a leading cause of morbidity and physical disability in people over 40 [3].

The gold standard treatment option for this aging society with greater functional demands [4] is total knee arthroplasty (TKA). Although there are numerous conservative therapy options available for mild-to-moderate OA [5], TKA is the most economical and successful treatment for treating advanced OA of the knee [6]. According to studies, after TKA, more than 90% of patients experience adequate pain relief and improved functional results, providing reassurance of its effectiveness [7]. In the United Kingdom, around 100,000 hip and knee arthroplasties are performed annually, while in the United States, they are about 400,000/year [8].

Patient-reported outcome measures (PROM) scores are a significant approach for assessing success post-arthroplasty since they evaluate the success of an intervention from the patient's perspective [9]. Joint-specific PROMs give the patient perspective on that specific joint's pain level and function. These metrics, such as the Oxford Knee Score (OKS), assess factors such as stiffness, pain, function, satisfaction, and quality of life, allowing you to quantify treatment success after surgery [10]. OKS has been validated in multiple studies and accepted in the orthopedic community as a reliable indicator of the outcome after TKA [11].

The primary goal of this study was to see the OKS in patients who underwent simultaneous bilateral knee replacement at six weeks and six months postoperatively and to see if there was a clinically significant difference in the mean OKS.

## Materials and methods

This prospective cross-sectional study was conducted at the Section of Orthopedic Surgery at Aga Khan University Hospital, a tertiary care center in Karachi, Pakistan. The institutional ethical review committee approved the study (approval number: 2023-7977-26781), ensuring the reliability and ethical conduct of the research.

The study's inclusion criteria involved patients who consented and underwent a simultaneous bilateral total knee arthroplasty procedure. Exclusion criteria included patients with a recent knee infection, as this could affect the surgical outcome, and extensor mechanism dysfunction who were candidates for revision knee arthroplasty, as their condition may require a different treatment approach.

A total of 50 patients were prospectively enrolled in the study from October 2023 to December 2023 and followed for the next six months. One patient was lost to follow-up.

Primary outcome measures were PROM and satisfaction score. PROM was accessed using the Oxford Knee Score (OKS). The OKS consists of 12 questions assessed on a Likert scale with values from 0 to 4. A summative score is then calculated where 48 is the best possible score (least symptomatic) and 0 is the worst possible score (most symptomatic). The minimal clinically significant difference (MCID) for the OKS is 5 points [12]. Satisfaction with the surgery was asked as a question at the six-month follow-up: "How would you describe the results of your procedure?" answered on a five-point scale from 1 (poor) to 5 (excellent). The OKS was collected preoperatively before the procedure, at six weeks and six months during regular outpatient visits. Essential demographic characteristics and surgical parameters for all included patients were recorded.

The surgical procedure used a cemented posterior stabilized total knee arthroplasty system (Zimmer), chosen for its proven efficacy and safety in knee replacement surgeries. In all the patients, a median parapatellar approach was utilized. Before the start of the procedure, all patients received a standard dose of 2 grams of injection cefazolin and 400 mg of injection ciprofloxacin Intravenously. Before the incision, injection tranexamic acid was administered intravenously at 10 mg/kg as a single dose. Pre and postoperatively, no mechanical thrombo-prophylaxis was utilized. All patients preoperatively received chemical prophylaxis only. The tourniquet at the proximal thigh was inflated at 300 mmHg and was not kept longer than 90 mins. A mechanical alignment technique was used with a median parapatellar approach. The duration of surgery was recorded as the time between the skin incision and closure of the incision. All the patients followed the same postoperative protocol in coalition with the physiotherapy team. They were taught how to do quadriceps-strengthening exercises and were ambulated full weight-bearing with a walker on the first postoperative day and this status continued till their clinic follow-up. Patients were typically discharged on the third postoperative day on oral aspirin 75 mg once daily. The patients were followed in the outpatient setting on the fourteenth postoperative day for removal of stitches, at four weeks for accessing quadriceps strength and allowing them a stick for ambulation in place of a walker, and then at three months and six months for regular follow-up.

Statistical analysis was performed using the Statistical Package for Social Sciences version for Windows, version 23 (IBM Corp., Armonk, NY). Quantitative variables were presented as mean ± standard deviation (S.D.), whereas qualitative variables were expressed as percentages. A p-value of <0.05 was considered significant. The student's paired or unpaired t-test was used to compare linear variables between groups. Dichotomous variables were assessed using the chi-square test. Spearman’s correlation test was used to assess the relationship between linear variables.

## Results

The total number of patients included in the study was 49. The mean age was 61.9 +/- 6.1. There were 42 (85.7%) females and 7 (14.3%) males. The mean BMI of our patients was 33.3 +/- 3.8, with the highest BMI being 43.0 and the lowest 27.0. In our study group, the major comorbidities recorded were diabetes mellitus, hypertension, ischemic heart disease, asthma, and hypothyroidism. Only 7 patients (14.2%) had no comorbidities, patients having 2 comorbidities or less were 23 (46.9%), and those having 3 comorbidities or more were 19 (38.8%). The radiographic Kellgren Lawrence Grading (KLG) for osteoarthritis was utilized on the preoperative weight-bearing X-rays for both knees. Thirty-eight (77.6%) patients were placed in Grade IV KLG, and 11 (22.4%) were placed in Grade III KLG. Forty (81.6%) patients had a preoperative diagnosis of primary osteoarthritis, whereas 9 (18.4%) had a diagnosis of rheumatoid arthritis (Table 1). All the patients presented with complaints of pain and progressively increasing varus deformity over the years. The mean duration of their complaints was 4.1 +/- 1.9 years.

**Table 1:** Demographic results

Parameters		Results, N (%)
Gender	Male	7 (14.3%)
	Female	42 (85.7%)
Kellgren Lawrence Grading	Grade III	11 (22.4%)
	Grade IV	38 (77.6%)
Primary Diagnosis	Osteoarthritis	40 (81.6%)
	Rheumatoid	9 (18.4%)
Early Complications	Superficial Infection	2 (4.1%)
	Atrial Fibrillation	1 (2.0%)
Satisfaction With Outcome	Excellent	25 (51.0%)
	Very Good	21 (42.9%)
	Good	3 (6.1%)
	Fair	0 (0%)
	Poor	0 (0%)

All patients underwent simultaneous bilateral knee replacement with cemented posterior stabilized knee prosthesis (Zimmer, Warsaw, IN, US). The mean duration of surgery was 160 +/- 10.1 mins from incision time to dressing of both the knee joints. The mean length of stay at the hospital was 5.8 +/- 0.9.

The early complications (< 6 weeks postoperatively) encountered were superficial wound site infection in 2 (4.1%) patients, which was managed with oral antibiotics in the outpatient setting, 1 (2.0%) of our patients developed atrial fibrillation on her sixth postoperative day, which was medically managed, but she remained stable and was discharged. There were no late complications (> six weeks to six months) such as aseptic loosening, dislocation, deep-vein thrombosis, or pulmonary embolism. None of the patients had symptoms consistent with aseptic loosening such as pain, dislocation, or alteration in the level of postoperative mobility. Postoperative radiographs were done during the six weeks and six months of follow-up visits. The radiographs demonstrated no evidence of aseptic loosening in any patient. None of the patients had readmission; the rates of inpatient mortality and overall mortality at six weeks and six months were 0%.

Mean OKS preoperatively was 12.6 +/- 3.5, with the lowest being 6 and the highest 21. At six weeks, the OKS showed improvement, with the mean being 20.6 +/- 3.0, the lowest score now being 16, and the highest 26. At six months postoperatively, there was a significant improvement in the OKS, with the mean now being 42.7 +/- 2.4, the lowest being 38, and the highest 48 (Table 2). At six weeks post-surgery, the mean improvement points in OKS score was 7.9 +/- 2.71, whereas at 6 months post-surgery, the mean improvement in OKS score was 30.1 +/- 3.6. This difference was significant (p-value=0.03) (Table 3).

**Table 2:** Oxford Knee Score (OKS) Sd - Standard Deviation

Oxford Knee Score		
	Mean +/- SD	OKS
Pre-operative	12.6 +/- 3.5	Lowest Score - 6
		Highest Score - 21
6 weeks postoperatively	20.6 +/- 3.0	Lowest Score - 16
		Highest Score - 26
6 months postoperatively	42.7 +/- 2.4	Lowest Score - 38
		Highest Score - 48

**Table 3:** Mean improvement points in Oxford Knee Score

Mean improvement points in Oxford Knee Score	
6 weeks	7.9
6 months	30.1

The Spearman correlation test was utilized to see if there was a significant association between age, comorbidities, BMI, and duration of surgery to improve the mean OKS score. However, the value was not found to be significant.

At the six-month follow-up, all patients were asked about their satisfaction with the procedure's outcome. Twenty-five (51.0%) reported the outcome as excellent, 21 (42.9%) reported it as very good, and 3 (6.1%) reported it as a good outcome. This was graded on a scale of 1 to 5 (5 being excellent, 4 very good, 3 good, 2 fair, 1 poor).

## Discussion

Our study had a majority female population of 85.7%, which was significantly higher than the reported numbers in the National Joint Registry of the UK, which reported that about 57% of the cases were performed in women [13]. In a review of literature worldwide, osteoarthritis is more common in females than in males [14]. In our local population, knee osteoarthritis is seen in about 3.6% of rural areas and 3.1-4.6% of urban areas of Northern Pakistan [15]. Data from the rest of Pakistan are scarce.

The mean age in our study was 61.9 (SD 6.1), which was lower as compared to Peterson et al., who reported a mean age of 65.6 (SD 11.5) [16], and Clement et al., who reported a mean age of 70.3 (SD 9.0) [17]. The lower mean age in our study group could be because the study was done in the country's most significant private healthcare hospital in the largest metropolitan city of Pakistan. Thus, the patients presenting to the hospital are more aware of their health problems and have easy access to excellent healthcare treatment options.

OKS is the PROM of choice to access the outcome of TKA for The National Joint Registry of England, Wales, and Northern Ireland [18]. The National Joint Registry uses OKS to assess the outcome of TKA at six months postoperatively [19]. Literature suggests that there is no optimal postoperative timepoint to access the outcome of TKA using the OKS. Hence, the OKS should represent the maximum outcome achieved by the patient group at the time of evaluation [12]. The recent Total or Partial Knee Arthroplasty Trial (TOPKAT) study accessed OKS at five years post-TKA; their results showed that it was not clinically different from one to five years in their two study groups [20]. A literature review showed a scarcity of data on the outcome of simultaneous bilateral total knee replacement with the OKS. In our study, we utilized the OKS after simultaneous bilateral total knee replacement at six weeks and then at six months.

The duration of surgery is an essential factor in TKA. It has been associated with a shorter hospital length of stay and a smaller risk of postoperative complications [21]. The mean duration of surgery for both knees in our study was 160 minutes, with a mean length of stay at the hospital of 5.8 days (SD 0.9). The operative time was comparable with Ryu et al.'s reported time of 153 minutes (SD 29.6) [22].

One (2%) of our patients had a systemic complication (atrial fibrillation), which is lower as compared to a study by Yoon et al., which showed systemic complications in 5%; 6 of their patients underwent simultaneous bilateral TKA [23]. In early complications, superficial infection was included, defined as an infection of the skin and subcutaneous tissue that resolves with antibiotics [23]; Yoon et al. reported superficial infection in three (2.5%) patients, comparable to our study. We had superficial infection in only two patients (4.1%). Our study group results encountered no late complications, showing zero mortality and no aseptic loosening and dislocation.

The study results show a statistically significant change in OKS from six weeks to six months. Our study had a mean preoperative OKS of 12.6 (SD 3.5), which was comparable to Clement et al. and Graham et al. which had mean preoperative OKS of 20.8 and 16.8 respectively [12,24]. In our study, the mean OKS at six weeks was 20.6 (SD 3.0), lower than that of Akhtar et al., who reported an OKS score of 31 at six weeks postoperatively [25]. At six months, OKS improved to 42.7 (SD 2.4), which was higher as compared to Clement et al. 35.2 (SD 9.0) [17].

The mean improvement point in OKS at six weeks was 7.9 (SD 2.7), which is comparable to the six weeks improvement of 7.0 reported by Akhtar et al [25]. The mean improved significantly at six months, with the OKS now at 30.1 (SD 3.6). This was significantly higher than in previous studies. Petersen et al. reported a mean six-month change in OKS of 16.5, and Beard et al.'s study reported a change of 14.7 [16,26].

Our study showed that the mean improvement points in OKS at six months were far greater than the mean improvement points in OKS at six weeks. Studies have found a significant mean improvement of 1 point [17] and 2 points [27] in the OKS between 6 and 12 months.

Strengths and limitations

The study's limitations are the small sample size and short follow-up of six months, as the OKS could change up to one year after the surgery [27]. The study's strength would be a prospective study and the Oxford Knee Score at six weeks post-surgery, which is not seen in the literature.

## Conclusions

Our study utilized the Oxford Knee Score (OKS) post-simultaneous bilateral total knee arthroplasty at six weeks and six months. Our results showed a clinically significant difference between the mean OKS postoperatively at the six-week and six-month timeline. There was also a remarkable increase in mean improvement points in OKS score at six months as compared to six weeks. With respect to the outcome of our study, we would like to assess the outcome of simultaneous bilateral knee arthroplasty with the OKS at six months postoperatively.
